# Receptor Pharmacogenomics: Deciphering Genetic Influence on Drug Response

**DOI:** 10.3390/ijms25179371

**Published:** 2024-08-29

**Authors:** Sorina Andreea Anghel, Cristina-Elena Dinu-Pirvu, Mihaela-Andreea Costache, Ana Maria Voiculescu, Mihaela Violeta Ghica, Valentina Anuța, Lăcrămioara Popa

**Affiliations:** 1 Department of Physical and Colloidal Chemistry, Faculty of Pharmacy, University of Medicine and Pharmacy “Carol Davila”, 6 Traian Vuia Str., 020956 Bucharest, Romania; sorina-andreea.anghel@rez.umfcd.ro (S.A.A.); mihaela-andreea.costache@rez.umfcd.ro (M.-A.C.); ana-maria.stiubeiu@rez.umfcd.ro (A.M.V.); mihaela.ghica@umfcd.ro (M.V.G.); valentina.anuta@umfcd.ro (V.A.); lacramioara.popa@umfcd.ro (L.P.); 2 Department of Molecular Cell Biology, Institute of Biochemistry, Splaiul Independentei 296, 060031 Bucharest, Romania; 3 Innovative Therapeutic Structures Research and Development Centre (InnoTher), “Carol Davila” University of Medicine and Pharmacy, 020956 Bucharest, Romania

**Keywords:** pharmacogenomics, precision medicine, polymorphism, G protein-coupled receptors, GPCR, receptor tyrosine kinase, RTK

## Abstract

The paradigm “one drug fits all” or “one dose fits all” will soon be challenged by pharmacogenetics research and application. Drug response—efficacy or safety—depends on interindividual variability. The current clinical practice does not include genetic screening as a routine procedure and does not account for genetic variation. Patients with the same illness receive the same treatment, yielding different responses. Integrating pharmacogenomics in therapy would provide critical information about how a patient will respond to a certain drug. Worldwide, great efforts are being made to achieve a personalized therapy-based approach. Nevertheless, a global harmonized guideline is still needed. Plasma membrane proteins, like receptor tyrosine kinase (RTK) and G protein-coupled receptors (GPCRs), are ubiquitously expressed, being involved in a diverse array of physiopathological processes. Over 30% of drugs approved by the FDA target GPCRs, reflecting the importance of assessing the genetic variability among individuals who are treated with these drugs. Pharmacogenomics of transmembrane protein receptors is a dynamic field with profound implications for precision medicine. Understanding genetic variations in these receptors provides a framework for optimizing drug therapies, minimizing adverse reactions, and advancing the paradigm of personalized healthcare.

## 1. Introduction

Over twenty years ago, the Human Genome Project, which provided the human genetic blueprint, was completed [1]. The genetic makeup of humans is 99.9% identical, while 0.1% accounts for genetic diversity resulting in phenotypic differences between individuals [2]. Each human being has a distinctive pattern of genetic variation that can impact disease susceptibility and drug response. The traditional medicine dogma is “one drug fits all” or “one dose fits all”, offering identical treatment to all patients that suffer from the same disease. However, the dosage can be individualized based on age or different comorbidities [3]. Using this approach, a great heterogeneity can be observed in drug response, giving rise to adverse reactions or treatment failure. In recent years, a more patient-focused type of medicine has surfaced, namely precision medicine. Modern tools such as technology-based omics, artificial intelligence, and big data analytics provide the interface that can depict individual variability and treat patients based on their uniqueness [4]. Pharmacogenetics and pharmacogenomics (PGx) are the pillars of precision medicine, attuning drug selection and dosage to each patient’s genetic profile [5]. Nevertheless, PGx testing is not a routine procedure. Since 2020, the U.S. Food and Drug Administration (FDA) issued a PGx information table that currently contains over 100 drug–biomarker pairs [6]. Moreover, for the last two decades, a powerful public database, PharmGKB (Pharmacogenomics Knowledgebase), has provided labeling information, clinical annotations, literature resources, and the pathways involved in gene–drug interactions [7,8,9]. Launched in 2009, the Clinical Pharmacogenetic Implementation Consortium (CPIC) has established clinical practice guidelines for PGx data, occasionally contrasting FDA drug labeling [10]. Thus, a global harmonized guideline needs to be implemented to translate PGx into clinical practice, increase the education of healthcare professionals, and offer patients personalized treatment.

PGx investigates the genetic variability in drug target genes (membrane receptors, enzymes, and ion channels) also known as pharmacodynamics genes as well as pharmacokinetic genes, which are genes involved in drug metabolism [11]. Despite significant efforts to gain a deeper understanding of all pharmacogenes, receptors PGx remains largely understudied. G protein-coupled receptors (GPCRs) and receptor tyrosine kinases (RTKs) are classical drug targets. GPCRs constitute the largest family of transmembrane proteins, transducing extracellular stimuli (hormones, neurotransmitters, and nutrients) into intracellular effects, thus controlling a plethora of bodily functions [12]. Although they serve as the most successful druggable protein family, as 35% of drugs target them, no GPCR variant is included in the drug labeling information [13]. Meanwhile, the genetic variation in a receptor tyrosine kinase, namely epidermal growth factor receptor (EGFR), whose overexpression was linked with cancer progression, is well defined in correlation with its inhibitors [13,14].

The integration of personalized medicine into common clinical practice has long been proposed, but challenges are yet to be defeated [15]. In this review, we will lay out the current status of PGx-informed pharmacotherapy regarding EGFR and emphasize the crucial significance of evaluating GPCR polymorphism in drug response.

## 2. Pharmacogenomics: Mechanism and Implementation

### 2.1. An Overview on Pharmacogenes Polymorphism

**Figure 1:**
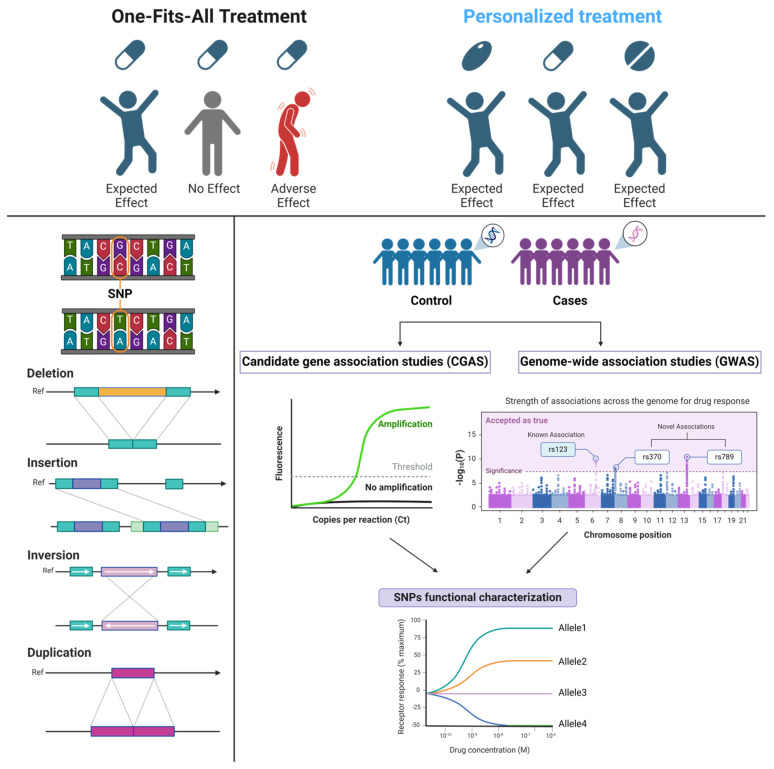
Pharmacogenetics overview. Traditional medicine operates based on the “one drug fits all” paradigm, treating all patients identically, while precision medicine employs “omics” methods, mainly pharmacogenomics, with the aim of providing patients with personalized treatment. Genetic differences between individuals may arise from different types of mutations, with the most prevalent being SNPs. In order to establish a correlation between a genetic variant and drug response, two methods are currently performed: candidate gene association studies and genome-wide association studies, each with its own advantages [21,22,23]. Adapted from “Personalized Medicine (AI vs. Traditional Techniques)” and “The Principle of a Genome-wide Association Study (GWAS)” template by BioRender.com. Retrieved from https://app.biorender.com/biorender-templates (accessed on 31 July 2024).

Pharmacogenomics (PGx) evaluates the influence of genetic variation on drug response. Although they are commonly used interchangeably, pharmacogenetics takes into account a single-gene effect, while PGx follows a more integrative approach by addressing the impact of the whole genome on drug treatment outcomes. Within the pharmacogenes, multiple types of genetic variations can arise due to single nucleotide polymorphisms (SNPs), indels (small insertions/deletions), inversions, duplications, or complex recombinations [16] (Figure 1). As a result, protein structure or function can be altered. The functional consequences of these mutations can result in an enhanced or novel protein activity in the case of gain-of-function (GoF) variants or in a diminished or non-existent activity owing to loss-of-function (LoF) variants [17]. Polymorphism in genes that encode drug-metabolizing enzymes or drug transport has a pharmacokinetic impact, while mutations in drug target genes (receptors, enzymes, and intracellular signaling proteins) result in an altered pharmacodynamics profile [18]. The most widely studied pharmacogenes are involved in drug metabolism, especially the well-known CYP450 family members, which are highly polymorphic, such as the CYP2D6 variants that have been thoroughly characterized [19]. On the contrary, pharmacodynamics drug–gene interactions are less understood [20]. Assessment of genetic variants that impact drug response relies on two approaches: the candidate gene approach (CGAS) or genome-wide association studies (GWAS). Both techniques explore the link between a phenotype (e.g., an adverse reaction or drug efficacy) and a genotype, although their study design is different. 

The first one requires pre-specified genes known to be involved in drug pharmacology (drug metabolism or drug action). For example, warfarin dosing depends on the genetic polymorphism of two candidate genes: the metabolizing enzyme, *CYP2C9*, and the drug target, *VKORC1* [24]. Since CGAS is a hypothesis-driven approach, it is more prone to bias if the wrong genes are chosen. Moreover, it is a self-limiting method because other important genes can be missed [25,26]. Meanwhile, GWAS employs a genome-wide screening strategy without the need for an a priori hypothesis. For instance, GWAS revealed that *SLCO1B1* polymorphism predicts the risk of statin-induced myopathy, leading to improved adverse reaction management for patients who have been prescribed statins [27]. GWAS can identify novel mutated genes responsible for the observed phenotype, making it a useful approach for common variants, while CGAS is a method for studying rare variants [23]. Comparisons between these genetic associations are reviewed here [28], favoring GWAS. It should be noted that GWAS have their own limitations: (a) they can identify many genetic variants and can be cumbersome to determine the causal genes; (b) large sample sizes are mandatory for achieving statistical significance; and (c) population stratification can generate a false association [22,29,30]. Published GWAS are usually focused on understanding diseases rather than drug response. Hence, further PGx GWAS are vital for discovering relevant therapeutic biomarkers and the pathways involved in drug action [30].

### 2.2. Clinical Implementation of Pharmacogenomics

Implementation of PGx in clinical practice is a slow process, regardless of its proven role in drug therapy. Few hospitals or clinics from all over the world are applying the PGx tools into their practice, performing routine genetic testing for their patients [31,32,33,34,35]. The resistance of healthcare professionals against genotype-specific therapy arises from multiple reasons: (1) an apparent insufficient clinical utility; (2) scarcity of cost-effectiveness studies; (3) lack of standardized genotyping tests for each drug–gene pair; (4) difficulty in interpreting PGx tests; and (5) absence of a specific guideline or a step-by-step procedure for patients with a mutated pharmacogene [20]. The gold standard of evidence-based medicine is represented by randomized controlled trials (RCTs). In traditional medicine, prescribing decisions regarding commonly used drugs can rely on disease-oriented evidence implemented by case series rather than RCTs [36,37]. In addition, RCTs are costly, patient-specific factors (age, polypharmacy, and comorbidities) may not be taken into account, and rare variants can be overlooked [38]. Thus, in the case of PGx-guided therapy, confirmation of clinical utility cannot rely only on RCTs; however, alternative forms of evidence (retrospective studies and meta-analysis) may be sufficient if they are properly designed and analyzed. The overall cost for PGx-based treatment has decreased in the past few years due to technological advances, e.g., from single gene testing to a multigene panel [39]. PGx tests are not yet reimbursed due to a lack of widespread cost-effectiveness studies, although recent data demonstrate a positive economic outcome when PGx testing is performed [40,41]. Lastly, PGx implementation is stalled as a consequence of an absent harmonized guideline. A global network of experts (researchers and clinicians) and patients would help propel PGx research through translation into clinical practice [42]. Since 2005, the FDA has recommended the use of PGx data in drug development for new drug applications, reinforcing the inclusion of PGx information in drug labeling [13,43,44]. Labeling sections can include indications and usage, dosage and administration, adverse reactions, clinical pharmacology, clinical studies, warnings, and precautions, and even boxed warnings can be used to display PGx biomarkers [13]. Moreover, since 2020, the FDA has issued a Table of Pharmacogenetic Associations categorizing different gene–drug pairs with an emphasis on drug-metabolizing enzymes, drug transporters, and gene variants that can produce certain adverse reactions [6]. Likewise, the European Medicines Agency published its first PGx guideline in 2012 covering the impact of pharmacokinetic modifications due to genetic variation in key proteins [45]. Currently, there are several organizations whose purpose is to formulate recommendations to facilitate PGx application in clinical practice, such as the Clinical Pharmacogenetics Implementation Consortium (CPIC), the Dutch Pharmacogenetics Working Group (DPWG), the Canadian Pharmacogenomics Network for Drug Safety (CPNDS), and the French National Network (Réseau) of Pharmacogenetics (RNPGx) [46]. Each assembly has a distinctive profile and a different approach. Until now, CPIC emitted 26 guidelines providing guidance only for patients that already have PGx data, unlike DPWG, CPNDS, and RNPGx, which advise performing genotype testing in daily clinical practice, with RNPGx offering specific clinical characteristics for when it is suitable to test [46]. Considering this discrepancy, an important question is being raised: when and how to test? Ideally, genotype testing would be performed before prescribing, providing a patient-oriented, personalized guide to drug selection and dosage, reducing adverse reactions, and avoiding ineffective treatments [47]. This approach is called pre-emptive PGx and shows great potential for achieving genotype-based therapy [48,49,50,51,52,53,54]. Usually, a panel with different variants associated with commonly used drugs is tested and the information obtained is recorded electronically, generating a digital PGx profile for each patient serving as a starting point for any following medical problems. However, PGx tests are largely unregulated, unstandardized, and difficult to interpret. Based on high-quality and consistent evidence, PGx tests need to have the same gene and allele content linked with recommendations regarding medication selection and dosing [55]. 

Initiatives and efforts to harmonize PGx specialization worldwide have been made. The Association of Molecular Pathology (AMP), an international non-profit scientific society, provides information regarding the choice of alleles to be included for testing. The CPIC and DPWG are standardizing terms for PGx test results, in line with the Pharmacogene Variation Consortium (PharmVar), which also offers a universal PGx nomenclature. Pharmacogenomics Clinical Annotation Tool (PharmCAT) uses CPIC variants and assigns the corresponding allele for PGx test results interpretation, generating a PGx report. PharmGKB is an important resource for healthcare providers that aids in spreading information about genetic variants and their implications in drug response [56,57]. Moreover, globally, in the last 20 years, PGx education has been incorporated in medical and pharmacy schools, although some refinement is still needed (e.g., more hours of PGx education to be adopted, PGx as an independent pharmacy specialty, a more interdisciplinary perspective for better integrating PGx information, and creating E-learning programs for low-developed countries) [58].

## 3. The Influence of GPCR Genetic Variation on Drug Response

### 3.1. GPCR Pharmacogenetics Overview

GPCRs are the largest superfamily of membrane proteins in the human genome with over 800 members [59]. Currently, approximately 400 drugs approved by the FDA mediate their effect via 108 GPCRs. Moreover, novel GPCRs, which are not a target for any approved drug, are being investigated in clinical trials [60]. Although functionally diverse, the 7TM receptors (seven-transmembrane receptors) share a common design: an extracellular N-terminus, a C-terminal cytoplasmic domain, and seven transmembrane domains (TM1-7) joined together by three extracellular loops (ECL1-3) and three intracellular loops (ICL1-3) [59]. Upon ligand binding, a conformational shift takes place that favors the interaction with heterotrimeric G proteins. The Gα subunit has an intrinsic GTPase activity and transits between a GDP-bound inactive state and a GTP-bound active one. The GTP-bound Gα dissociates from the Gβγ subunit, triggering further cellular signaling. Based on the Gα subunit, G proteins are designated as Gα_s_, Gα_i/o_, Gα_q/11_, and Gα_12/13_ and regulate the activity of adenylyl cyclase, phospholipase C, or RhoGEFs [61]. Another GPCR interacting partner is represented by β-arrestins, which mediate desensitization and receptor internalization. Furthermore, β-arrestin-dependent, G protein-signaling has been described [62], but it is still under investigation regarding the G protein implication [63].

**Figure 2:**
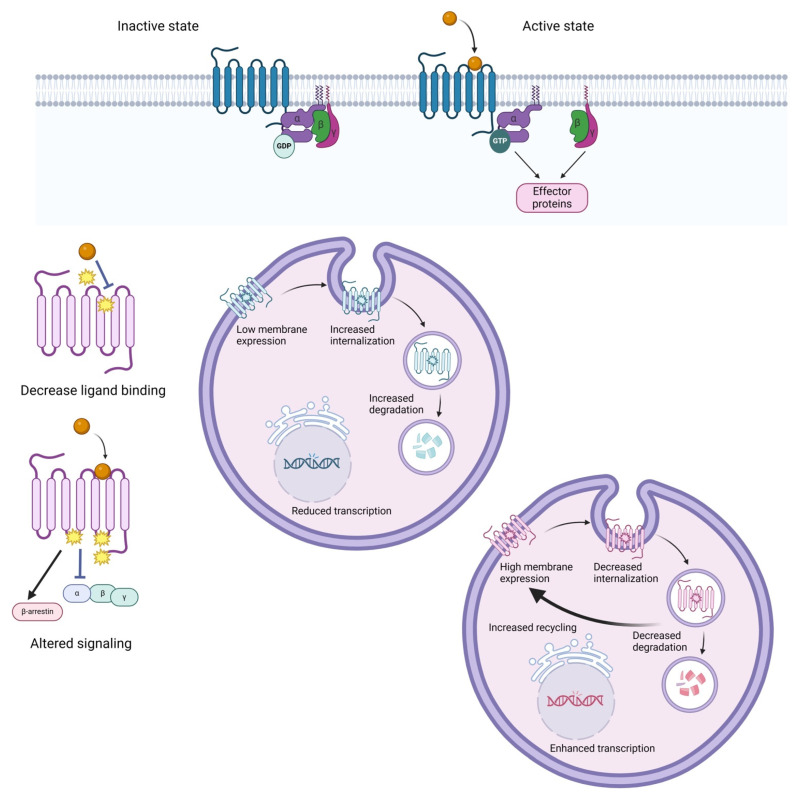
GPCR signaling and functional consequences of mutated forms. Upon ligand binding, GPCRs function like GEFs (guanine -exchange factors) favoring the exchange of GDP-bound Gα subunit to GTP-bound Gα subunits, initiating the signaling cascade. Mutations present in the ligand binding pocket (different for every GPCR class [72]) will halt the ligand–receptor interaction. Genetic variants affecting the G protein/β-arrestin binding domains (ICL2, ICL3, and C-tail [61,73]) can result in blocked or biased signaling. Moreover, some mutated receptor forms can present altered trafficking and disturbed desensitization, internalization, and degradation mechanisms [74]. Adapted from “Cell Endocytosis (Layout)” by BioRender.com. Retrieved from https://app.biorender.com/biorender-templates (accessed on 31 July 2024).

GPCR mutations were previously linked with disease occurrence, namely mutated rhodopsin receptor with retinitis pigmentosa [64], the calcium-sensing receptor with hypercalcemic syndromes [65], arginine vasopressin receptor 2 with nephrogenic diabetes insipidus [66], and many others [67,68,69,70]. Fewer studies exist regarding drug response. GPCR drug targets can be largely affected by missense mutations, followed by LoF variations that comprise nonsense mutations resulting in introducing a stop codon or frameshift mutations and copy number variations (deletions/duplications) [71]. The resulting functional implications can include decreased ligand binding, modified G protein selectivity or altered expression, localization, trafficking, or desensitization (Figure 2) [71]. 

The most targeted GPCRs, based on the number of approved drugs, are the adrenergic receptors, followed by the histamine receptors, serotonin, muscarinic, dopamine, and opioid receptors [60]. β-blockers, opioid analgesics, selective serotonin reuptake inhibitors, and second-generation antipsychotics that regulate dopaminergic and serotoninergic systems are among the most prescribed drugs in the US [75]. In the following subsections, the PGx of these receptors in correlation with drug reaction will be described and notable genetic variants are summarized in Table 1.

### 3.2. Antihypertension Therapy and β1-Adrenoreceptor Polymorphism

Adrenergic receptors are responsible for the regulation of the sympathetic nervous system, responding to endogenous catecholamines: adrenaline and noradrenaline. Ubiquitously expressed throughout the body, the nine receptor subtypes (α1A, α1B, α1D, α2A, α2B, α2C, β1, β2, and β3) have a multitude of indications as drug targets [97], but available PGx data focus mainly on β1-adrenergic blockade. Gene variants associated with dysregulated β1-adrenergic signaling are rs1801252:S49G (N-terminus) and rs1801253:R389G (helix 8) [98,99]. Greater reduction in blood pressure by metoprolol [76] or carvedilol [77] was observed for individuals with an R389R genotype than those who carried the R389G variant. Moreover, the S49R389/S49R389 diplotype can serve as a response predictor [76]. The *ADRB1* Ser49-Arg389 haplotype was associated with an increased risk of all-cause death, especially in individuals treated with verapamil, contrary to atenolol-treated individuals, indicating a protective role of the β1-blocker [100]. However, atenolol produced a higher risk of adverse cardiovascular events in the case of individuals who have the *ADRB1* S49G genotype [79].

### 3.3. Analgesics Treatment and µ_1_-Opioid Receptor Polymorphism

The µ_1_-opioid receptor (MOP) belongs to the class of opioid receptors that binds encephalin and β-endorphin-mediating analgesics effects. The classical µ_1_-opioid receptor agonist is morphine, but more potent synthetic ligands have been developed, such as fentanyl and derivatives serving as first-line therapy in moderate to severe pain management [101]. The most common SNP in the *OPM1* gene is rs1799971:N40D, causing the loss of an N-glycosylation site [102], and being associated with a high sensitivity to opioids and increased susceptibility to opiate abuse [83,103].

### 3.4. Antipsychotic Drug Treatment and Dopamine/Serotonin Receptor Polymorphism

The D1 class of dopamine receptors includes D1 and D5 receptors, while the D2 class of dopamine receptors is comprised of D2, D3, and receptors. Antipsychotic drugs, regardless of their generation (typical—first generation; atypical—second generation) act primarily as D2 receptor antagonists. Intensive D2 receptor blockade, being linked with adverse events, is predominant in the first generation of antipsychotics. The second generation of antipsychotics also blocks the serotonin receptors (5HT_1A_, 5HT_2A/2C_, 5HT_6_, and 5HT_7_) [104]. We focused mainly on atypical antipsychotics that act as dopamine–serotonin antagonists (clozapine, risperidone, olanzapine, and aripiprazole).

*DRD1* variants, rs4532 (A>48G) and rs5326 (G>94A), located in the 5′ UTR region are frequently studied [105]. Previously, the *DRD1* rs4532 variant was associated with decreased clozapine response [106,107], but a meta-analysis showed a lack of correlation [108]. In the case of *DRD2* polymorphisms, the most notable genetic variant is rs1799732: −141C Ins/Del, which is associated with reduced antipsychotic effects (Del allele compared with Ins/Ins genotype) [86]. Risperidone treatment response may be altered by genetic variants of DRD2, such as rs1801028:S311C, 141C Ins/Del (rs1799732), T939C (rs6275), rs6277, and TaqID [87]. While the rs6277 variant in DRD2 is associated with decreased response to ariprazole [109], the rs2514218 variant was correlated with better treatment response to aripiprazole or risperidone [110]. The rs6280:S9G variant is the most studied variant of the *DRD3* gene, affecting the N-terminal extracellular domain [111]. This variant can serve as a predictor for olanzapine [112] or clozapine responses [113]. 

Atypical antipsychotic responses are similarly disturbed by mutated serotoninergic receptors. Risperidone efficacy was associated with rs6699866 in *HTR6*, while polymorphic *HTR2C*, *HTR3D,* and *HTR5A* have insignificant effects [114]. The *HTR3B* variant rs1176744:Y129S has an increased affinity for serotonin, with induction of dopamine signaling that opposes the antipsychotic effect, thus being associated with a decrease in treatment response [115]. During clozapine or olanzapine treatment, the *HTR2A* haplotype (rs6311:1438A, rs6312:783A, rs6313:102T, and rs6314:1354T) is correlated with a lower risk of developing metabolic abnormalities [116]. 

### 3.5. Antidepressants and Serotonin Receptors

Selective serotonin reuptake inhibitors (SSRIs) are the most prescribed antidepressants, indicated for major depressive disorder and anxiety-related disorders. Inhibition of the serotonin transporter (SERT) in the presynaptic cleft, results in reduced serotonin uptake that can bind to 5HT1A autoreceptors [117,118]. Moreover, the SSRI vilazodone acts as a partial agonist for 5HT1A [119]. Other antidepressants that modulate the serotoninergic transmission are serotonin–noradrenaline reuptake inhibitors (SNRIs).

The *HTR1A* polymorphism in antidepressant response is still controversial; a few studies found no correlation [120,121], while others showed a better antidepressant response for patients expressing rs1364043, rs10042486, and rs6295 genetic variants when treated with milnacipran, fluvoxamine, fluoxetine, or paroxetine [91]. The rs6295 allele can also serve as a treatment predictor for citalopram response in older patients [122]. In the case of *HTR2A*, two common variants located in the promoter region have been widely studied, namely rs6311 and rs6313 [123]. These variants have been linked with higher treatment response; an increased risk of adverse reactions was associated with rs6311, while the rs6313 variant appears to have a protective effect [124]. A couple of studies reported no interdependency between the rs6311 and the antidepressant response [125,126,127], while for the rs6313 variant, a significant association was found [126]. 

## 4. EGFR, a PGx-Guided Therapy Model

EGFR, known as ErbB1/HER1, belongs to EGFR family together with ErbB2/HER2, ErbB3/HER3, and ErbB4/HER4. We chose *EGFR* as an example for receptor PGx-informed therapy because (1) it is expressed in different cancer types, (2) its mutational landscape is well characterized, (3) aberrantly expressed EGFR is targeted by the majority of tyrosine kinase inhibitors (TKIs), and (4) for a drug target, it has a significant amount of PGx data [128,129,130,131].

**Figure 3:**
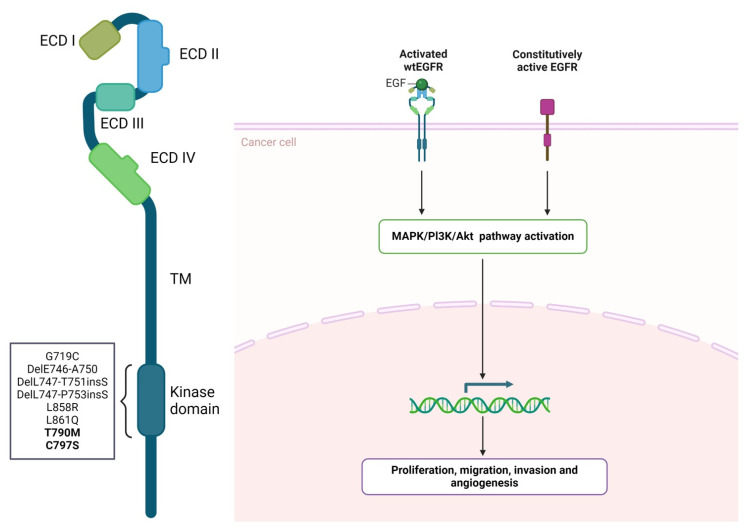
EGFR mutations and signaling in cancer. Kinase-activating mutations and EGFR overexpression are responsible for EGFR signaling amplification, which defines the typical cancer cell: uncontrolled proliferation and survival. EGFR-targeted therapy improves patient prognosis. Nonetheless, secondary mutations can emerge (T790M and C797S, in bold), giving rise to resistant cancer cells. Adapted from “EGFR and EGFRvIII Roles in Cancer Biology” by BioRender.com. Retrieved from https://app.biorender.com/biorender-templates, (accessed on 31 July 2024) [134,135].

EGFR signaling plays a pivotal role in regulating essential biological processes, including cell proliferation, differentiation, migration, adhesion, and survival [132]. As a membrane-spanning receptor, EGFR connects extracellular stimuli to the intracellular signaling pathways. Structurally, EGFR features four extracellular domains (I, II, III, and IV), one transmembrane domain, one juxtamembrane domain, and one tyrosine kinase domain, followed by a flexible regulatory C-terminus (Figure 3) [133]. Under resting conditions, EGFR exists as a monomer. Upon ligand binding to the extracellular domain, dimerization of the receptor occurs, followed by autophosphorylation and initiation of a downstream signaling cascade, namely the rat sarcoma (Ras)/mitogen-activated protein kinase (MAPK) and phosphoinositide 3-kinase (PI3K)/protein kinase B (PKB) pathways [132].

Renowned as an oncogene, the aberrantly expressed EGFR represents a malignancy hallmark, being more prevalent in lung, colorectal, and brain cancers [136]. Usually, *EGFR* oncogenicity is characterized by overexpression, gene amplification, or activating mutations [137]. The majority of the mutations occur within the *EGFR* exons 18 to 21, which encode a portion of the EGFR kinase domain (Figure 3) [138,139,140]. The purpose of EGFR-targeted therapy is to detect EGFR-positive cancers and existent mutations and to combat frequently acquired drug resistance. Deviant EGFR is managed by two treatment directions, tyrosine kinase inhibitors (TKIs) and monoclonal antibodies (Mabs) (as described in Table 2, which contains information from the FDA Table of Pharmacogenomic Biomarkers in Drug Labeling). Among the approved EGFR-TKIs, gefitinib and erlotinib belong to the first generation of EGFR inhibitors. They work by binding reversibly to the TK domain of the EGFR, blocking ATP binding, thereby halting EGFR activation and cellular proliferation. Afatinib and dacomitinib are members of the second generation of EGFR-TKIs, binding irreversibly to the EGFR, thus inhibiting its kinase activity, while osimertinib binds covalently to the cysteine residue in the EGFR [141,142]. Mobocertinib is designed to target EGFR exon 20 insertions [143]. Monoclonal antibodies (cetuximab, panitumumab, and amivantamab) bind to the extracellular domain III of EGFR, promoting ligand blocking, receptor–antibody complex internalization, and degradation [144]. Of note, kinase-activating mutations cannot disturb the activity of these antibodies since their binding site is in the extracellular domain.

According to PharmGKB, the *EGFR* is a Very Important Pharmacogene (VIP). Currently, on the PharmGKB database, there are 21 clinical annotations regarding *EGFR*, 13 of them involving the interaction between EGFR inhibitors and their target (Table 3) [8,9].

The FDA Table of Pharmacogenomic Biomarkers in Drug Labeling offers a perspective on available EGFR-targeted drugs and their PGx information, being indication-oriented (using an FDA-approved test to identify the specific drug targeted mutation for treatment). The clinical annotations from PharmGKB resemble the FDA Table of Pharmacogenetic Associations, offering information about the interaction between a genetic variant and a drug. Each pair contains the “Details” section where a specific allele is linked with an increased or decreased response, adverse reaction, or even with the likelihood of acquired resistance. Furthermore, the level of evidence is provided, with level 3 being the most assigned. In contrast, the FDA Table of Pharmacogenetic Associations contains gene–drug interactions that impact drug metabolism and have sufficient scientific evidence. At the moment, *EGFR* is not included in this table. Therefore, healthcare providers should take into account all available resources to guide and treat each patient individually based on their genetic makeup and to monitor therapy outcomes.

The major indication of EGFR-targeted therapy is represented by non-small-cell lung cancer (NSCLC). In NSCLC, the effectiveness of conventional chemotherapy treatments does not exceed 40%, being also associated with a high degree of toxicity and poor prognosis. Abnormal EGFR signaling contributes to the oncogenic phenotype in over half of NSCLC patients. TKIs interact with the aberrantly expressed receptor, significantly increasing the survival rate of patients and exceeding classical chemotherapy response levels in NSCLC patients [146]. Of all *EGFR* mutations, 45% are deletions or insertions (amino-acid residues 747 to 752) of exon 19, with the most common being delE746_A750. Additionally, the exon 21 point mutation L858R accounts for roughly 40% of *EGFR* mutations. The third most common type (10% of all *EGFR* mutations) consists of in-frame insertions and indels in exon 20 [147]. Exons 18, 19, and 21 mutations are sensitivity predictors of EGFR-TKI therapy, while mutations in exon 20 are generally resistant [148]. The emergence of *EGFR* T790M and C797S mutations (exon 20) has led to rapid resistance development against first-, second-, and third-generation EGFR-TKIs. The C797S mutation specifically hinders irreversible EGFR inhibitors’ ability to bind covalently to the kinase. Additionally, the rise of *EGFR* double and triple mutations poses challenges to the therapeutic efficacy of EGFR-TKIs, emphasizing the continuous necessity for potent new inhibitors [141].

While promising results are limited for other types of cancer besides NSCLC, erlotinib in combination with gemcitabine received FDA approval for use in the treatment of locally advanced, unresectable, or metastatic pancreatic cancer on 2 November 2005. In pancreatic cancer, EGFR overexpression is correlated with advanced disease, poor survival, and metastasis. Combining EGFR inhibitors with chemotherapy results in the inhibition of tumor-induced angiogenesis, promotion of tumor cell apoptosis, and tumor regression in xenograft models [149]. The non-frameshift deletion in exon 19 and the L858R point mutation in exon 21 are known as *EGFR*-sensitive mutations and respond well to EGFR-TKIs [150]. However, these mutations are rare in pancreatic ductal adenocarcinoma (PDAC) [151]. Similarly, in colorectal cancer, the main contributor to poor prognosis is EGFR overexpression [152]. Hence, blocking EGFR signaling is the ideal approach. Cetuximab and panitumumab are both indicated for EGFR-expressing, RAS wild-type metastatic colorectal cancer, serving as monotherapy or in combination with chemotherapy [153]. Additionally, cetuximab is also indicated for head and neck cancer in combination with radiation therapy, antineoplastic agents, or alone for recurrent disease or metastasis [154]. However, in this case, cetuximab therapy has a poor response rate, regardless of high *EGFR* gene amplification. Less than 5% of head and neck cancers present *EGFR* mutations, making this cancer type particularly difficult to treat [155]. Mutations or genetic variations in *EGFR* alone cannot account for all the differences in cancer patients’ responses to EGFR-targeted treatments. Beyond the *EGFR* genotype, genetic variances in other components of the signaling pathway downstream of EGFR or in other receptor tyrosine kinase pathways can significantly affect the effectiveness of specific EGFR-targeted therapies [156,157,158], emphasizing the personalized medicine approach of treating individual patients based on their particular physiology and needs.

## 5. Conclusions

In the digital era, personalized medicine can be achieved in order to provide patients with tailored therapies. By employing PGx tools, each patient can be treated based on their unique genetic makeup, reducing the risk of adverse reactions or treatment failure. However, it is still unclear when the PGx tests are recommended and how to apply and interpret them. Despite great efforts, the implementation of PGx-informed therapy is still incomplete. Moreover, the healthcare authorities (FDA and EMA) issued guidelines regarding only pharmacokinetic genetic variants due to a lack of sufficient evidence. One problem may be the inconsistency noticed between different studies that analyzed the same gene–drug pair. Thus, standardization is also an absolute requirement to further advance the field of PGx. In the case of commonly prescribed drugs, genetic variants that affect drug response are still understudied. Here, we used an RTK EGFR as a prototype for emphasizing the crucial role of determining receptors’ genetic influence on drug response. As more than one-third of approved drugs target GPCRs, a complete mutational landscape must be generated. We focused only on genetic variants that affect targets, without including studies evaluating the downstream signaling partners. Cellular signaling functions as an interconnected machinery. Hence, the net drug response may be a consequence of different altered proteins from the same pathway or from intersected pathways.

## Figures and Tables

**Table 1:** Known GPCR variants that affect drug efficacy and/or safety. LABA, long-acting β-agonists; * first generation: chlorpromazine and haloperidol; second generation: clozapine, risperidone, olanzapine, and aripiprazole.

Gene	Variant	Ligands	Effects	Ref.
*ADRB1*	rs1801253:R389G	Metoprolol Carvedilol Bisoprolol	↓ Antihypertensive response	[76,77,78]
	rs1801252:S49G	Atenolol	↑ Adverse cardiovascular events	[79]
*ADRB2*	rs1042713:R16G	LABA	↑ Risk of asthma exacerbation	[80]
	rs1800888:T164I	Salbutamol	↓ Ligand binding	[81]
*ADRA2C*	rs11269124: Del 322–325	Clonidine	↑ Efficacy	[82]
*OPM1*	rs1799971:N40D	Endogenous opioids Buprenorphine	↑ β-endorphin binding affinity ↓ Expression ↓ Efficacy for buprenorphine	[83,84,85]
*DRD2*	rs1799732 *DRD2* PROM −141C Ins/Del	Antipsychotics *	↓ Efficacy ↑ Weight gain	[86,87,88]
*HTR1A*	rs6295	Clozapine	↑ Efficacy	[89]
		Fluoxetine Paroxetine Milnacipran	↑ Efficacy	[90,91]
*HTR2A*	rs6313	Risperidone	↑ Adverse cardiovascular events	[92]
	rs6314	Clozapine	↑ Efficacy	[93]
		Olanzapine	↑ Efficacy	[94]
	rs6311	Escitalopram	↑Memory loss	[95]
	rs7997012	Antidepressants	↑ Treatment outcome ↑ Remission	[96]

**Table 2:** TKIs and monoclonal antibodies that target EGFR based on the FDA Table of Pharmacogenomic Biomarkers in Drug Labeling [13]. NSCLC, non-small-cell lung cancer; CRC, colorectal cancer; and SCCHN, squamous cell carcinoma of the head and neck.

Drug	Drug Class	Labeling Sections	Type of Cancer	Targeted Mutation
Erlotinib	TKI (I)	Indications and usage, dosage and administration, adverse reactions, and clinical studies	NSCLC Pancreatic cancer	Exon 19 deletion Exon 21 L858R
Gefitinib	TKI (I)	Indications and usage, dosage and administration, and clinical studies	NSCLC	Exon 19 deletion Exon 21 L858R
Afatinib	TKI (II)	Indications and usage, dosage and administration, adverse reactions, and clinical studies	NSCLC	Exon 19 deletion Exon 21 L858R
Dacomitinib	TKI (II)	Indications and usage, dosage, and administration, adverse reactions, use in specific populations, and clinical studies	NSCLC	Exon 19 deletion Exon 21 L858R
Osimertinib	TKI (III)	Indications and usage, dosage and administration, adverse reactions, and clinical studies	NSCLC	Exon 19 deletion Exon 21 L858R T790M
Mobocertinib	TKI	Indications and usage, dosage and administration, adverse reactions, and clinical studies	NSCLC	Exon 20 insertion
Amivantamab-vmjw	Mab	Indications and usage, dosage and administration, adverse reactions, and clinical studies	NSCLC	Exon 20 insertion
Cetuximab	Mab	Indications and usage, dosage and administration, adverse reactions, and clinical studies	CRC SCCHN	
Panitumumab	Mab	Adverse reactions, clinical pharmacology, and clinical studies	CRC	

**Table 3:** Clinical EGFR-related annotations from the PharmGKB database [145].

Drug	*EGFR* Variant	Level of Evidence	Pharmacology	Phenotype
Gefitinib	rs121434568	1A	Efficacy	Carcinoma, NSCLC
	rs121434569	2B	Efficacy	Carcinoma, NSCLC, drug resistance
	rs2293347	3	Efficacy	Carcinoma, NSCLC
	rs712829	3	Efficacy	Neoplasms
	rs11568315	3	Efficacy	Carcinoma, NSCLC
Erlotinib	rs121434569	2B	Efficacy	Adenocarcinoma, carcinoma, NSCLC, drug resistance, lung neoplasms
	rs712829	3	Efficacy	Neoplasms
	rs712829	3	Toxicity	Neoplasms
	rs2227983	3	Toxicity	Carcinoma, NSCLC, colorectal neoplasms, neoplasms, pancreatic neoplasms
Cetuximab	rs2227983	3	Efficacy	Head and neck neoplasms
	rs712829	3	Efficacy	Colorectal neoplasms
	rs712830	3	Toxicity	Colorectal neoplasms
Panitumumab	rs712829	3	Efficacy	Colorectal neoplasms

## Data Availability

No new data were created or analyzed in this study. Data sharing is not applicable to this article.
